# Evaluation of Antioxidant Status of Two *Limoniastrum* Species Growing Wild in Tunisian Salty Lands

**DOI:** 10.3390/antiox2030122

**Published:** 2013-08-02

**Authors:** Mohamed Debouba, Sami Zouari, Nacim Zouari

**Affiliations:** 1 High Institute of Applied Biology of Medenine, Environmental Sciences Department, University of Gabes, route El Jorf-Km 22.5, Medenine 4119, Tunisia; E-Mail: znacim2002@yahoo.fr; 2 Arid Regions Institute, Medenine 4119, Tunisia; E-Mail: samiaz2004@yahoo.fr

**Keywords:** *Limoniastrum guyonianum*, *Limoniastrum monopetalum*, antioxidant, metabolites, DPPH, ferrous ions chelating activity, reducing power, TBARS

## Abstract

We aim to highlight the differential antioxidant status of *Limoniastrum guyonianum* and *Limoniastrum monopetalum* in relation to their respective chemical and location characteristics. Metabolite analysis revealed similar contents in phenolic, flavonoïds, sugars and chlorophyll in the two species’ leaves. Higher amounts of proline (Pro), carotenoïds (Carot), sodium (Na) and potassium (K) were measured in *L. monopetalum* leaves relative to *L. guyonianum* ones. While the two *Limoniastrum* species have similar free radical DPPH (2,2-diphenyl-1-picrylhydrazyl) scavenging activity, *L. guyonianum* showed more than two-fold higher ferrous ions chelating activity relative to *L. monopetalum*. However, highest reducing power activity was observed in *L. monopetalum*. Thiobarbituric acid-reactive substances (TBARS) determination indicated that *L. monopetalum* behave better lipid membrane integrity relative to *L. guyonianum*. These findings suggested that the lesser stressful state of *L. monopetalum* was related to higher metabolites accumulation and reducing capacity compared to *L. guyonianum*.

## 1. Introduction

Spontaneous native plants of North Africa are known to have ecological, pastoral and medicinal importance. In particular, perennial halophytes are able to colonize regions with extreme climate and soil characteristics, such as salinity, drought and heavy metals contamination [1,2]. Besides, valuable biotechnological uses are being attributed to halophytes in bioremediation [3], pharmaceutical and industrial futures [4].

The obligate halophytes *Limoniastrum monopetalum* and *L.* *guyonianum*, belonging to family Plumbaginaceae, have long constituted very dense populations; they became endangered and are now broken up into discontinuous islands, due to urban invasion. *Limoniastrum monopetalum* is a shrub from sebkhas and coastal saline depressions [5]. *Limoniastrum* *guyonianum* grows wild on salty arid land and desert [5]. *Limoniastrum* galls are used in southern Tunisia for tanning leather and dyeing hair. The infusion of galls and leaf is used against infectious or parasitic disease causing a painful and bloody diarrhea [5]. *Limoniastrum monopetalum* and *Limoniastrum guyonianum* have pastoral importance, especially for camels during the winter [6].

At an ecological level, the species of *Limoniastrum* genus have a very effective role in the stabilization of coastal dunes. Moreover, the formation of galls in the shoots of these species constitutes an ecological habitat of a large number of insects such as *Lepidoptera* and *Hymenoptera* (*Oecocecis guyonella*, *Acalyptris limoniastri*) [7].

While huge data are given in the literature about crop plants responses to salt abiotic stress, little is available about spontaneous species responses when growing wild in their natural ecosystems. The present work aims to investigate the *in situ* antioxidant status of two *Limoniastrum* species growing wild in Tunisian salty lands: *L.* *monopetalum* and *L.* *guyonianum*. The obtained changes are discussed focusing on the relative chemical composition of each *Limoniastrum* species.

## 2. Experimental Section

### 2.1. Plant Sampling

Plants were collected from two different sites (Table 1).

**Table 1:** Main characteristics of harvest sites.

Species	Region name	Coordinates UTM	Temperature (°C)	Rainfall (mm/year)
*L. guyonianum*	Oued el Fja (Medenine) Salt marsh	Latitude: 33°29′33.93″N Longitude: 10°38′24.70″E	Max: 36.8 Min: 6.2	144
*L. monopetalum*	Hassi Jerbi (Zarzis) seashore	Latitude: 33°38′13.34″N Longitude: 11°0′22.49″E	Max: 32 Min: 6	200

### 2.2. Ion Analysis

Inorganic ions were extracted from dry materials with 0.5 N H_2_SO_4_ at room temperature for 48 h. Sodium and potassium were analysed by flame emission using spectrophotometer (Eppendorf, Netheler-Hinz, GmbH Hamburg, Germany).

### 2.3. Methanolic Extract Preparation and Analysis

Samples of 10 g of dried shoots from each *Limoniastrum* species were finely ground using a homogenizer and extracted with 80% methanol at room temperature for 24 h. Each mixture was then filtered through Whatman No. 42 filter paper to remove the debris, and the extracts were then evaporated using a rotary evaporator. The crude extracts were suspended in 80% methanol.

#### 2.3.1. Polyphenols Contents

Phenolic content was determined in methanolic extract by the Folin-Ciocalteu method Heimler *et al.* [8] using gallic acid (GA) as phenolic standards, and expressing the results as Gallic acid equivalents per g dry weight (mg GA/g DW).

#### 2.3.2. Flavonoids Contents

A slightly modified version of the spectrophotometric method [9] was used to determine the flavonoids contents in methanolic extract. An aliquot (0.5 mL) was taken in a test tube and 3 mL of distilled water and 0.3 mL of 5% NaNO_2_ were added. The solution was mixed well and allowed to stand at room temperature for 5 min. To this solution, 0.6 mL of 10% AlCl_3_ was added. After 6 min, 2 mL of 1 M NaOH was added to the test tube. The solution was then diluted with distilled water to make the final volume up to 10 mL. The absorbance was read at 510 nm. Flavonoids content was expressed as mg Quercetin*/*g dry weight (mg QC*/*g DW).

#### 2.3.3. DPPH Radical-Scavenging Assay

DPPH scavenging activity of methanolic extract was measured by the slightly modified spectrophotometric method of Brand-Williams *et al.* [10]. A solution of DPPH in methanol was freshly prepared. A 3 mL aliquot of this solution was mixed with 100 mL of the samples at varying concentrations (50–250 μg/mL). The solutions in the test tubes were shaken well and incubated in the dark for 15 min at room temperature. The decrease in absorbance was measured at 517 nm. The antiradical activity was expressed as IC50 (μg*/*mL), the antiradical dose required to cause a 50% inhibition. The percentage inhibition of the radicals due to the antioxidant property of the extracts was calculated using the formula (1):

Inhibition (%) = [(*A*_control_ − *A*_sample_) × 100]/*A*_control_ (1)

#### 2.3.4. Ferrous Ions Chelating Activity

The chelating of ferrous ions by plant methanolic extract was estimated as described by Zhao *et al.* [11]. Briefly, different concentrations of plant part extracts were added to a 0.05 mL FeCl_2_ 4H_2_O solution (2 mmol/L) and left for incubation at room temperature for 5 min. After the reaction was initiated by adding 0.1 mL of ferrozine (5 mmol/L), the mixture was adjusted to 3 mL with deionised water, shaken vigorously, and left standing at room temperature for 10 min. Absorbance of the solution was then measured spectrophotometrically at 562 nm (Anthelie Advanced 2, SECOMAN). Analyses were run in triplicates. The percentage of inhibition of ferrozine-Fe^2+^ complex formation was calculated using the formula (2) given bellow:

Metal chelating effect (%) = (*A*_0_ − *A*_1_) × 100/*A*_0_ (2)

where *A*_0_ is the absorbance of the control, and *A*_1_ is the absorbance in the presence of the sample extracts or standard. Results were expressed as EC_50_: efficient concentration corresponding to 50% ferrous iron chelating.

#### 2.3.5. Ferric-Reducing Activity

The reducing power of the methanolic extract was determined by the method of Yildirim *et al.* [12]. Sample solutions (0.5 mL) with different concentrations were mixed with 1.25 mL of 0.2 M phosphate buffer (pH 6.6) and 1.25 mL of potassium ferricyanide solution (10 g/L). The mixtures were incubated for 30 min at 50 °C. After incubation, 1.25 mL of trichloroacetic acid (100 g/L) was added and the reaction mixtures were centrifuged for 10 min at 3000 *g*. A 1.25 mL aliquot of the supernatant from each sample was mixed with 1.25 mL distilled water and 0.25 mL of ferric chloride solution (1.0 g/L) in a test tube. After a 10 min reaction time, the absorbance was measured at 700 nm. EC_50_ value (μg*/*mL) is the effective concentration at which the absorbance was 0.5 for reducing power and was obtained from linear regression analysis.

### 2.4. Lipid Peroxide Determination

The level of lipid peroxidation in leaves was assessed in terms of malonydialdehyde (MDA) content by thiobarbituric acid (TBA), as recommended by Heath and Parcker [13], with minor modifications following Dhindsa *et al.* [14]. Fresh samples were homogenized in trichloroacetic acid (TCA) (0.1% p/v), then centrifuged at 8000 *g* for 15 min. The reaction mixture contains 1 mL of supernatant added with 4 mL 20% TCA containing TBA (0.5% p/v). A blank tube was made by using 1 mL of 20% TCA instead of supernatant in the reaction mixture. The mixture was heated in a water bath shaker at 95 °C for 30 min and quickly cooled in an ice bath. The absorbance was measured at 532 nm, and the value for non-specific absorption at 600 nm was subtracted. The MDA content was calculated using its extinction coefficient ε = 155 mM^−1^·cm^−1^.

### 2.5. Proline Contents

Proline was determined by the method of Bates *et al.* [15]. Plant tissue (0.5 g) was homogenized with 5 mL of 3% aqueous sulfosalicylic acid and then the homogenate was centrifuged at 14,000 *g* for 2 min. Two milliliters of acid ninhydrin and glacial acetic acid were added into 2 mL of the homogenate in a test tube. The mixture was then incubated at 100 °C for 1 h, after which the reaction was stopped by placing the test tube in an ice bath. Four milliliters of toluene were added to each test tube and vortexed for 15–20 s. The organic and inorganic phases were separated, and the absorbance at 520 nm of the organic toluene phase containing the chromophore was used to quantity the amount of proline.

### 2.6. Chlorophyll and Carotenoids Determination

Chlorophyll (Chl) contents were determined by the method of Arnon [16]. The absorbance of a sample was read at 460 nm, 645 nm and 663 nm, then contents of Chl a, Chl b and carotenoids (cart) were calculated using the formulas of MacKinney [17].

## 3. Results and Discussion

### 3.1. Metabolites Contents

Both *Limoniastrum* species have similar phenolic and flavonoids contents. They showed four to five-fold higher phenolic contents relative to flavonoids ones (Table 2). With reference to literature, polyphenols levels measured in these *Limoniastrum* species were higher than those found in *L. monopetalum* collected from inferior semi arid bioclimatic stage [4]. Compared to other halophytes such as *Mesembryanthemum* *edule* grown on arid bioclimatic stage, the two *Limoniastrum* species have similar flavonoids levels but phenolics ones were about three-fold higher [4].

*L. monopetalum* was significantly more enriched in the other analyzed metabolites compared to *L. guyonianum*. Proline, potassium, carotenoids and sugars were respectively about 25%, 32%, 36%, 28% and 36% higher in *L. monopetalum* relative to *L. guyonianum* (Table 2). It is known that the two *Limoniastrum* species are obligate halophyte and grow only in saline soils (Table 1). We found that *L. monopetalum* accumulated larger sodium levels than *L. guyonianum* (Table 2). Sodium contents in *L. monopetalum* tissues were also higher when compared to an obligate halophyte *Sesuvium portulacastrum* grown on saline medium [2].

**Table 2:** Changes in metabolites contents in the leaves of *Limoniastrum guyonianum* and *Limoniastrum monopetalum*. Each data is the average of three replications ± SD. Means sharing at least one same letter are not significantly different according to Tukey test at *p* < 0.05.

Metabolites	*Limoniastrum guyonianum*	*Limoniastrum monopetalum*
Phenolic content (mg GA/g DW)	217.82 ± 14.38a	225.22 ± 8.10a
Flavonoids contents (mg QC/g DW)	50.20 ± 5.23 b	42.26 ± 5.70b
Proline (μg/g DW)	521.43 ±22c	642.86 ± 19.20d
Sugars (mg/g DW)	28.70 ±3e	38.00 ±3.22g
Carotenoids (mg/g FW)	0.22 ± 0.07h	0.30 ± 0.03i
Chl (mg/g FW)	0.10 ±0.003j	0.11±0.005j
Sodium (μmol/g DW)	4889.62 ± 29k	11358.21±41l
Potassium (μmol/g DW)	788.34 ±14m	1011.14 ±18n

It is noticeable that in contrast to most common plants, the leaves of these two *Limoniastrum* species showed more than two-fold higher carotenoids contents compared to chlorophyll ones (Table 2). It seems that in such extreme environment conditions, carotenoids play a protective role for chlorophyll molecules that ensure the vital photosynthetic activity.

Accumulation of mineral ions (sodium, chloride and potassium) and organic compounds (proline and sugars) into vacuoles of studied *Limoniastrum* species may insure osmotic adjustments [18]. Tolerance of *Atriplex nummularia* to salinity was attributed to its ability to keep Na^+^ and Cl^−^ away from enzymes and sequester them within organelles, together with an effective osmotic adjustment [19].

Polyphenols and flavonoids accumulated in *Limoniastrum* species leaves constitute one of the most diverse and widespread group of natural compounds. These compounds possess a broad spectrum of biological activities including antioxidant and radical scavenging properties [20,21,22].

### 3.2. Scavenging of DPPH Radicals

DPPH assay is one of the most widely used methods for screening the antioxidant activity of plant extracts. The assay is based on the measurements of the antioxidants ability to scavenge the stable nitrogen-centered free radical DPPH. Our results clearly indicate the potential of *Limoniastrum* extracts in scavenging free radicals. Calculation of extract concentration (μg/mL) inducing 50% inhibition of DPPH radical showed that there was no significant difference between the two *Limoniastrum* species (Table 3).

**Table 3:** Antioxidant activities of *Limoniastrum guyonianum* and *Limoniastrum monopetalum* leaves. Data are the average of three replications ± SD. Means sharing at least one same letter are not significantly different according to Tukey test at *p* < 0.05.

Antioxidant activities	*L. guyonianum*	*L. monopetalum*
DPPH scavenging activity (IC_50_ μg/mL)	14.90 ± 3a	17.14 ± 2.4a
Ferrous ions chelating activity (IC_50_ μg/mL)	191.63 ± 12b	90.15 ± 9c
Reducing power (EC_50_ μg/mL)	109.5 ± 11d	42.33± 5e

### 3.3. Ferrous Ions Chelating Activity

It is known that iron at ferrous state (Fe^2+^) is a powerful prooxidant among the various species of metal ions. Comparing the IC_50_ (the extract concentration (μg/mL) inducing 50% ferrous ion chelating), we stated that *L. guyonianum* exhibited at least two-fold higher ferrous ions chelating activity relative to *L. monopetalum* (Table 3).

### 3.4. Ferric Reducing Power

The reducing power of both *Limoniastrum* leaves gradually increased with extract concentration (data not shown). When calculating the EC_50_ (the extract concentration at which the absorbance was 0.5 at 700 nm), we found that *L. monopetalum* extract were more efficient in reducing the Fe^3+^/ferricyanide complex relative to *L. guyonianum* (Table 3).

While the two *Limoniastrum* species showed comparable phenolic and flavonoids contents, they exhibited differences towards antioxidants capacities. For instance, our results showed that *L.* *guyonianum* have the highest anti-metallic activity; conversely, *L.* *monopetalum* have strongest ferric reducing power (Table 3). This differential antioxidant behavior suggested that *L.* *monopetalum* was enriched in phenolic compounds that are more efficient in breaking Fe^3+^/ferricyanide complex than in chelating metals. In fact, the efficiency of phenolic compounds as anti-radicals or reductones depends on the number of hydroxyl groups bonded to the aromatic ring, the site of bonding and mutual position of hydroxyls in the aromatic ring [23]. On the other hand, *L.* *guyonianum* polyphenols seemed to be slightly more efficient in scavenging the DPPH free radicals (Table 3). It is known that only flavonoids with a certain structure and particularly, hydroxyl position in the molecule can act as proton donating and show radical scavenging activity [24]. Furthermore, the extracts are very complex mixtures of many different compounds with distinct activities [21,24].

### 3.5. Malonydialdehyde Content (MDA)

The products of lipid peroxidation which react with thiobarbituric acid are termed TBARS (Thiobarbituric acid reacting substances) and are characteristic of aldehydes, mainly malonydialdehyde (MDA), products of monohydroperoxydation and secondary oxidation of lipids. The content of MDA is considered as a marker to estimate the extent of oxidation of membrane lipids and damage caused by stress conditions.

**Figure 1:**
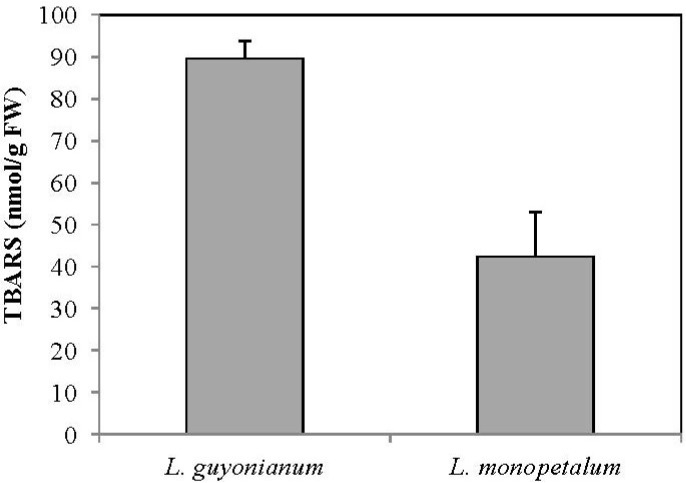
Thiobarbituric acid-reactive substances (TBARS) in leaves of *Limoniastrum* *guyonianum* and *Limoniastrum monopetalum*. Data are means of at least three replicates ± SD at *p* ≤ 0.05.

According to Figure 1, MDA contents were two-fold higher in *L. guyonianum* than *L. monopetalum* ones. This result suggested that *L. monopetalum* behave like efficient mechanisms that ensure membrane integrity and prevent cell toxicity against oxidative stress.

In the present experiment, measurement of MDA revealed that *L.* *guyonianum* was more affected by its environmental conditions than *L.* *monopetalum* (Figure 1). It seems that the lesser lipid damage in *L.* *monopetalum* compared to *L.* *guyonianum* was related to (i) the stronger reducing power in *L.* *monopetalum* leaf extracts, (ii) the higher carotenoids contents that are known to play an efficient antioxidant role, namely for chlorophyll molecules to sustain sufficient photosynthetic activity (Table 2), (iii) an adequate mineral nutrition (Table 2), and (iv) the moderate environment conditions at seashore compared to inland salty marsh where *L. guyonianum* was collected (drought, salinity, high temperature) (Table 1).

Thus, the difference between the two species towards antioxidant status and metabolites accumulation appeared to be determined by physiological plasticity and the climate zone conditions where they were widely grown [25]. Such variability could be of great importance in understanding the regulation of a natural antioxidants biosynthesis. Previous studies have shown that the amount of plant polyphenols and antioxidant activity depends on soil proprieties (temperature, salinity, light intensity) [26].

Besides phenolic compounds, the antioxidant capacity can be attributed to other metabolite such as proline (Table 2). It has been shown that proline, in addition to its osmotic role, is a protector of cytoplasmic proteins [27]. It can help to mitigate the effect of reactive oxygen species (ROS) on the enzyme proteins [28]. It should also be noted that the ability to conquer oxidative stress is also ensured by powerful antioxidant systems including enzymatic components and volatile compounds [29].

## 4. Conclusions

Our results show that *L.* *monopetalum* and *L.* *guyonianum* leaves exhibited differential behavior in term of mineral accumulation and antioxidant capacities. We stated that antioxidant pattern was not regulated exclusively by polyphenols contents; rather polyphenols with specific structure, proline and plant species could be involved. Further analysis is needed to indentify enzymatic antioxidant systems conferring *Limoniastrum* species tolerance to extreme environment.
